# Supplementary material for: Long-range ferrimagnetic order in a two-dimensional supramolecular Kondo lattice
Source: Nat Commun. 2017 May 22;8:15388. doi: 10.1038/ncomms15388 (PMC5458152; doi:10.1038/ncomms15388)
Supplement: Supplementary Information — Supplementary Figures, Supplementary Notes, Supplementary Tables and Supplementary References [file ncomms15388-s1.pdf]

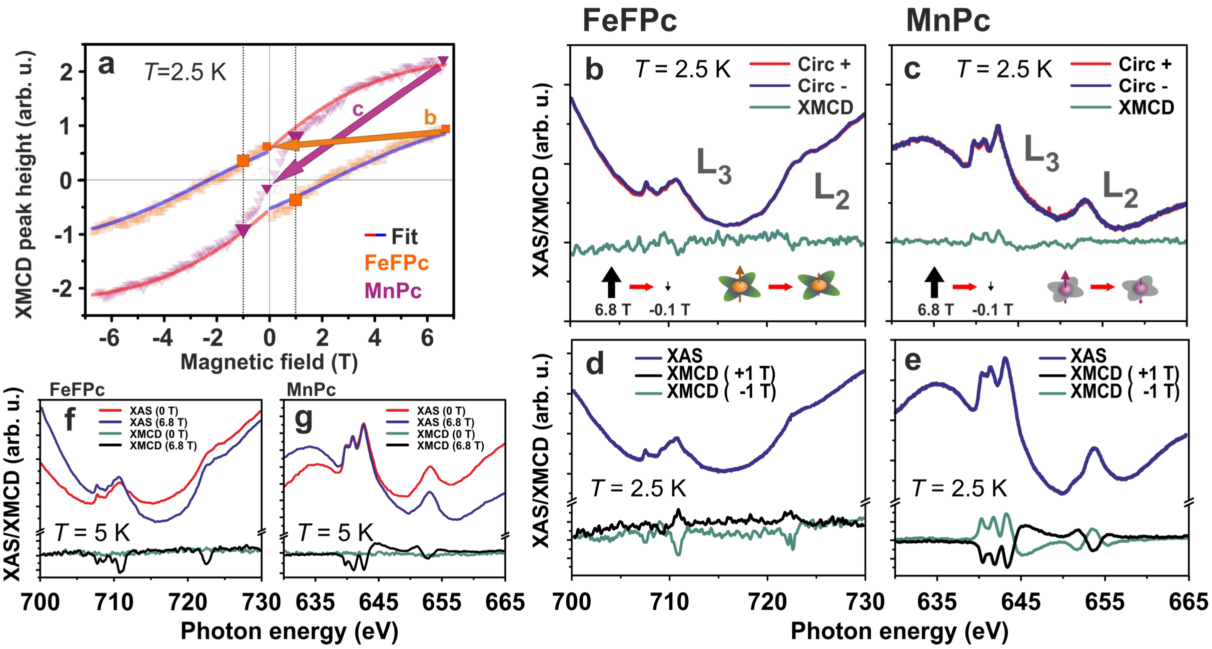

Supplementary Figure 1 | Normal incidence XAS/XMCD measurements of the FeFPc+MnPc/Au(111) system. (**a**) XMCD peak height vs *B* curves recorded for MnPc and FeFPc at *T* = 2.5 K in normal incidence. Arrows show the protocol used to unravel the magnetic coercivity of the FeFPc and MnPc molecules within the 2D array. A magnetic field of *B* = 6.8 T was applied initially to saturate the system and subsequently the field was continuously ramped down to -0.1 T, during which the XAS/XMCD spectra were recorded. The XAS and XMCD spectra of FeFPc (**b**) reveal that the direction of the magnetic moments did not change. Conversely, the XMCD spectra of the MnPc species (**c**) show that these have changed their XMCD sign in comparison to that observed at *B* = 6.8 T. The insets of panels **b** and **c** highlight the orientation of external magnetic field (black arrows) and orientation of the magnetic moments with respect to the field (sketches of the molecules). (**d**) and (**e**) XAS/XMCD spectra measured in magnetic fields of *B* = ±1 T and at *T* = 2.5 K confirm the antiferromagnetic coupling between the MnPc and FeFPc magnetic moments. The dotted lines in panel **a** are used to highlight the magnetic fields (*B* = ±1 T) at which the XAS/XMCD spectra of panels **d** and **e** were measured. Panels (**f**) and (**g**) present XMCD measurements acquired at *T* = 5 K for *B* = 0 T and *B* = 6.8 T evidencing no remanent magnetisation on either of the molecules.

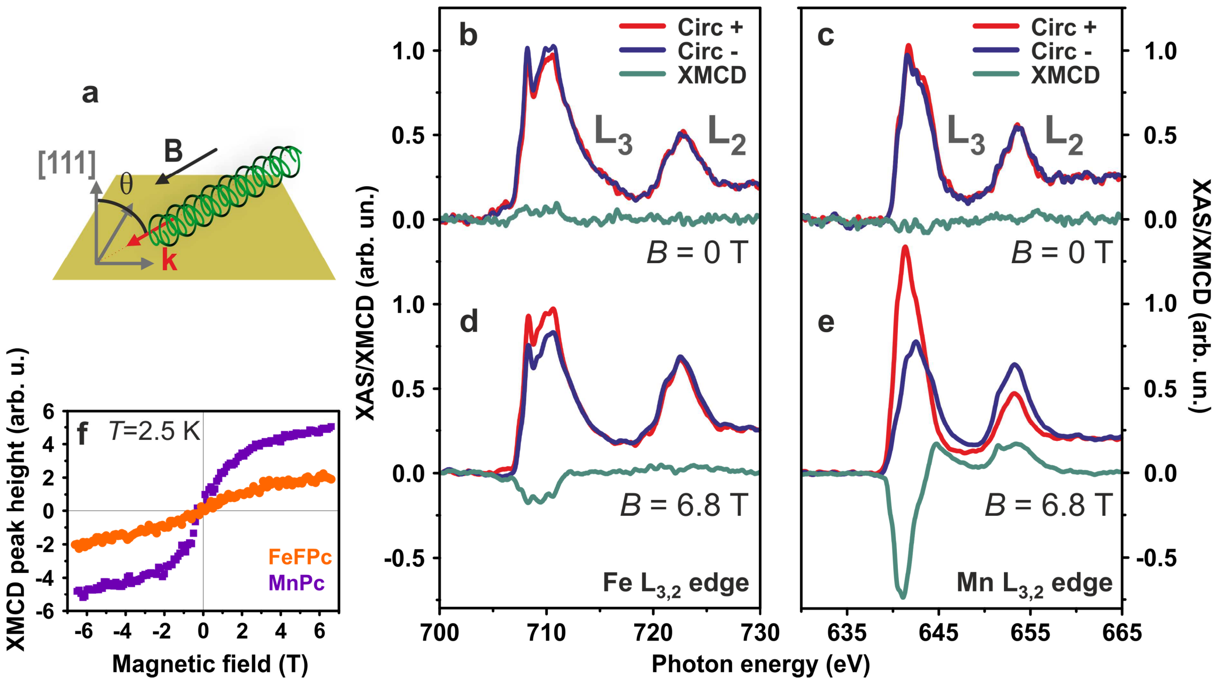

Supplementary Figure 2 | XAS and XMCD spectra acquired on the FeFPc+MnPc array on Au(111). (**a**) Sketch of the grazing incidence measurement geometry, where the surface normal [111] and the **k**-vector of the incoming X-rays are at an angle $\theta = 70$ degrees. In this configuration the XAS/XMCD probes predominantly the in-plane components of the magnetic moments, with a part of the out-of-plane components contributing to the overall signal[1,2]. The external magnetic field **B** is parallel to the **k**-vector of the incoming X-rays. (**b,c**) XAS and XMCD measured for FeFPc and MnPc at the $L_{3,2}$ edges in zero field and (**d,e**) in an applied field of 6.8 T, at T = 2.5 K. The XMCD spectra acquired on MnPc molecules in zero applied external magnetic field reveal no dichroic signal in **c**, which suggests that the remanent magnetic moments of the MnPc molecules are fully aligned along the out-of-plane direction. The XMCD spectrum acquired at the Fe $L_{3,2}$ edge of FeFPc molecules in zero field resolves a small remanent magnetisation in **b**. (**f**) Element-specific XMCD peak height vs B curves measured on the Fe and Mn ions reveal no remanent magnetisation. The curves have been recorded according to the protocol described in the Methods section.

Supplementary Note 1.

To investigate the magnetic coercivity of our 2D spin array, we performed XAS/XMCD measurements at T = 2.5 K with the magnetic field continuously ramped down from 6.8 T to -0.1 T (see orange and violet arrows in **Supplementary Figure 1a**). As shown in **Supplementary Figure 1b,c** the sign of the XMCD of the FeFPc molecules did not change, whilst that of the MnPc molecules did, in comparison to XMCD spectra acquired at 6.8 T (cf. **Figure 1c**). The observed behaviour of the XMCD signals suggests that the coercivity of the spin array is smaller than 0.1 T. In case the system would exhibit a coercivity higher than 0.1 T, one would observe an opposite behaviour, i.e. the FeFPc molecules changing the XMCD sign, whilst the MnPc species do not. Note that due to the spiky behaviour of the total electron yield around zero field and the continuous field ramping with the need for binning into certain B-field bins and interpolation, there is an increased noise in XMCD peak height vs B curves around zero field.

Applying the mean field approximation to the bipartite Ising model and assuming only a nearest neighbour interaction between MnPc and FeFPc molecules, we can extract the strength of the indirect exchange coupling from the XMCD peak height vs B curves. At B = 2 T the magnetic moments of FeFPc molecules are equal to zero, which means that the Zeeman energy and exchange coupling are of equal strength. The FeFPc molecules are surrounded with 4 MnPc species, therefore the effective magnetic field induced by the 4 MnPc molecules is equal to $4J_{\mathrm{Fe-Mn}}\langle S_{\mathrm{Mn}}\rangle$, where $\langle S_{\mathrm{Mn}}\rangle$ = 1 μ_B (i.e. estimated at B = 2 T). Hence, we extract the exchange coupling strength $J_{\mathrm{Fe-Mn}}$ to be about 0.12 meV, which is significantly lower than the Kondo temperature of about 10 K (see below). Note that in our calculation we neglected anisotropy terms. Knowing the strength of the indirect exchange coupling we estimate an ordering temperature of T_C ~ 3.7 K. This value is in line with our XMCD measurements performed at B = 0 T and T = 5 K, shown in **Supplementary Figure 1f,g,** in which we cannot resolve a detectable XMCD signal.

In **Supplementary Figure 2** we present XAS/XMCD data acquired on the chessboard FeFPc+MnPc array on Au(111) in grazing incidence geometry, where the XMCD probes predominantly the in-plane components of the magnetic moments, and partly the out-of-plane components contributing to the overall signal[1,2]. The XMCD spectrum acquired at the Fe $L_{3,2}$ edge of FeFPc molecules resolves a small remanent magnetisation (**Supplementary Fig. 2b**), which is attributed to the out-of-plane component. Applying an external magnetic field of B = 6.8 T leads to alignment of both magnetic moments along the field. A sum-rule analysis applied on the XMCD spectra of both molecules (see **Supplementary Table 1**) resolves that in the field-aligned state (B = 6.8 T), the total magnetic moments are larger in grazing incidence than in the normal incidence geometry. This finding is in line with a previous report on thin films of FePc, which were found to exhibit easy-plane magnetic anisotropy[3]. A salient difference between these two cases, FePc films and the here-studied supramolecular layer, is discussed below.

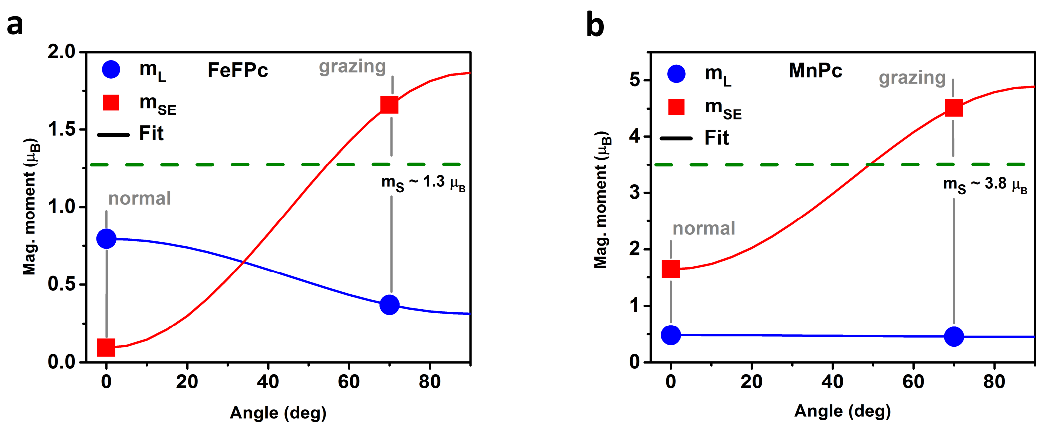

Supplementary Figure 3 | Angular dependence of the magnetic moments measured at *B* = 6.8 T. (**a**) Angular dependence of the orbital and effective spin magnetic moment of FeFPc molecules, and (**b**) of MnPc molecules obtained in the field-aligned state. The measured data points (symbols) are fitted to supplementary equations (3) and (4) for the orbital and spin effective moments, respectively, to obtain the isotropic spin moments. The dashed lines depict the extracted respective isotropic spin moments.

Supplementary Note 2.

In order to extract absolute values of the spin and orbital magnetic moments of the central metal ions of the MnPc and FeFPc molecules, the acquired XMCD data have been processed with the sum-rule analysis[4,5]. The orbital magnetic moment has been extracted using the following equation,

$$m_{\mathrm{L}}[\mu_{\mathrm{B}}] = -\frac{4}{3}\frac{\int_{\mathrm{L_3+L_2}}(\mathrm{C^+}-\mathrm{C^-})dE}{\int_{\mathrm{L_3+L_2}}(\mathrm{C^+}+\mathrm{C^-})dE}\langle N_{\mathrm{h}}\rangle \qquad (1)$$

and the effective spin magnetic moment has been obtained from

$$m_{\mathrm{SE}}\,[\mu_{\mathrm{B}}] = m_{\mathrm{S}} + 7m_{\mathrm{T}} = -2\frac{\int_{\mathrm{L_3}}(\mathrm{C^+}-\mathrm{C^-})dE - 2\int_{\mathrm{L_2}}(\mathrm{C^+}-\mathrm{C^-})dE}{\int_{\mathrm{L_3+L_2}}(\mathrm{C^+}+\mathrm{C^-})dE}\langle N_{\mathrm{h}}\rangle, \qquad (2)$$

where the integration of the circularly polarised XA spectra ($\mathrm{C^+}$,$\mathrm{C^-}$) is over the energy range defined by the respective L_3 and L_2 edges, <N_h> stands for average number of holes at a metal ion (<N_h> = 4 for FeFPc and <N_h> = 5 for MnPc). The term m_S stands for the spin magnetic moment and m_T is the intra-atomic dipolar moment resulting from the spin-quadrupolar anisotropy[6].

The square planar symmetry of the phthalocyanine ligand at the metal ion causes both the effective spin and orbital magnetic moments to be angle dependent (see **Supplementary Figure 3**) with the following dependency[9],

$$m_{\mathrm{L}}(\theta) = m_{\mathrm{L}}^{\mathrm{OP}} cos^2(\theta) + m_{\mathrm{L}}^{\mathrm{IP}} sin^2(\theta) \qquad (3)$$

$$m_{\mathrm{SE}}(\theta) = m_{\mathrm{S}} + 7m_{\mathrm{T}}^{\mathrm{IP}}\left(2cos^2(\theta) - sin^2(\theta)\right) \qquad (4)$$

where $m_{\mathrm{L}}^{\mathrm{OP}}$ and $m_{\mathrm{L}}^{\mathrm{IP}}$ are the out-of-plane and in-plane components of the orbital magnetic moment, respectively, and θ is the angle between the **k**-vector of the incoming X-rays and the surface normal. The term $m_{\mathrm{T}}^{\mathrm{IP}}$ in supplementary equation (4) stands for the in-plane component of the dipolar m_{T} term, and $2m_{\mathrm{T}}^{\mathrm{IP}} + m_{\mathrm{T}}^{\mathrm{OP}} = 0$. We can thus extract the spin magnetic moment m_{S} of the MnPc and FeFPc molecules as ~ 3.8 μ_{B} and ~ -1.3 μ_{B}, suggesting a spin *S* ~ 3/2 – 2 for MnPc and *S* ~ 1/2 for FeFPc. The reduced value of the FeFPc spin moment (as compared to the *S* = 1 of a free FeFPc molecule) is consistent with the underscreened Kondo effect suggested previously for FePc molecules on Au(111)[10].

In **Supplementary Table 1** we present the thus-obtained values of the magnetic moments of the FeFPc and MnPc molecules both in normal and grazing incidence.

Supplementary Table 1. Values of the measured magnetic moments of the FeFPc and MnPc molecules both in normal and grazing incidence and with and without applied external magnetic field, extracted from a sum-rule analysis. The negative sign of the magnetic moments on the FeFPc molecules means an opposite direction of the XMCD signal as compared to that of the MnPc species. Since the sum-rule analysis tends to overestimate the effective spin moment of Mn^{2+} ions by 33%[7,8]. The values of the Mn magnetic moments have therefore been corrected by multiplying them by a factor 0.7.

B = 0 T				*B* = 6.8 T		
m_{SE} [μ_{B}]	m_{L} [μ_{B}]	$m_{\mathrm{SE}} + m_{\mathrm{L}}$ [μ_{B}]		m_{SE} [μ_{B}]	m_{L} [μ_{B}]	$m_{\mathrm{SE}} + m_{\mathrm{L}}$ [μ_{B}]
0.07	0.28	0.35	MnPc normal	1.65	0.48	2.13
0	0	0	MnPc grazing	4.5	0.45	4.95
0.14	-0.39	-0.25	FeFPc normal	0.1	0.79	0.89
-0.24	-0.16	-0.4	FeFPc grazing	1.66	0.37	2.03

Supplementary Figure 3 reveals an unusual angular dependence of the magnetic moments. In the field aligned state the size of the orbital moment of FeFPc decreases from a higher value observed in normal incidence to a smaller one measured in grazing incidence geometry. On the other hand, the angle dependence of the effective spin moment shows an opposite behaviour with a higher value in grazing incidence and smaller one in normal incidence. A somewhat similar behaviour is observed for MnPc, however with the change in the orbital moment being comparably smaller. Notably, the behaviour found here for FeFPc on Au(111) is different from that observed previously for thin films of FePc[3] and for the FePc/graphene/Ir system[11], where both effective spin moment and orbital moment increased with the incident angle resolving an easy-plane anisotropy. Considering that the orbital moment is pivotal to the magnetic anisotropy[12] our data reveal an out-of-plane easy axis for FeFPc and MnPc in the supramolecular layer.

In a similar, but more pronounced way the effective spin and orbital moments behave in remanence (see **Supplementary Table 1**). The moments measured for MnPc are Ising-like, i.e. only non-zero in the out-of-plane direction. For FeFPc the orbital moment is again considerably larger in the out-of-plane direction, consistent with an out-of-plane easy axis in the remanent magnetic state. Interestingly, our measurements evidence a different behaviour of the magnetic anisotropy for the two-dimensional supramolecular layer as compared to thick films of FePc[3].

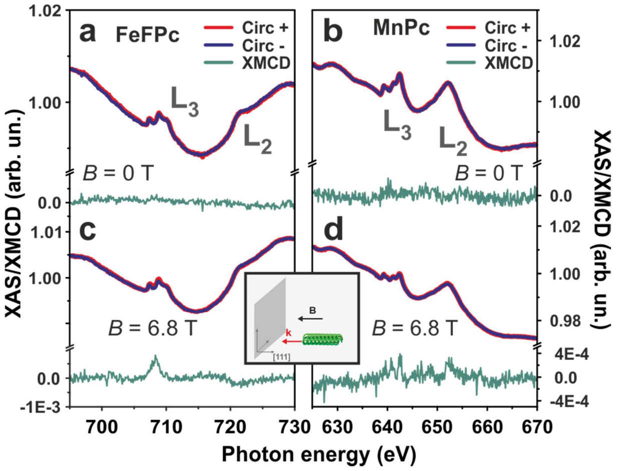

Supplementary Figure 4 | XAS/XMCD spectra acquired on the FeFPc+MnPc/Ag(111) system. (**a**) Measured XAS/XMCD spectra of FeFPc molecules in the supramolecular array self-assembled on a Ag(111) substrate in zero field (B = 0 T) shows zero remanent magnetisation. Similarly, the XAS/XMCD spectra of MnPc (**b**) molecules in the same supramolecular array exhibit zero remanent magnetisation in B = 0 T. The data have been acquired in normal incidence geometry with the **k**-vector of the incoming X-rays and external magnetic field aligned along the direction of the surface normal, i.e. the [111] axis (see inset). (**c**,**d**) In the applied external magnetic field (B = 6.8 T) the magnetic moments align along the direction of the magnetic field. Note: the spectra are superimposed on a background caused by the vicinity of the Ag $M_{3,2,1}$ absorption edges. All measurements were performed at T = 2.5 K.

Supplementary Note 3.

In this Supplementary Note we report XAS/XMCD spectra acquired on the supramolecular FeFPc+MnPc array assembled on Ag(111) measured at T = 2.5 K in normal incidence geometry.

The supramolecular array was deposited on a Ag(111) substrate to investigate the role of the substrate on the inter-molecular magnetic coupling. In **Supplementary Figure 4** we present XAS/XMCD data acquired on this system in normal incidence geometry. This is the same geometry where the supramolecular array assembled on the Au(111) substrate shows ferrimagnetic order in remanence. However, the dichroic spectra acquired on both molecules at zero magnetic field show zero magnetization, i.e. no remanence. Next, we apply an external magnetic field (B = 6.8 T) leading to alignment of the molecular magnetic moments along the field direction. We thereby confirm the pivotal role of the Au(111) substrate in mediating the indirect magnetic exchange coupling between MnPc and FeFPc molecules.

The Au(111) substrate hosts spin-split surface states with the following Fermi-wavevectors: 1.67 and 1.92 nm^{-1}, which are much larger than the Fermi-wavevector of Ag(111) surface states (k_F = 0.8 nm^{-1}) (supplementary ref. 13). Simulating the oscillatory behaviour of the RKKY coupling using the formula $J \propto \sin(2k_\mathrm{F}d)/(2k_\mathrm{F}d)^2$ (supplementary ref. 14), with the aforementioned Fermi wavevectors of the surface states and d being the distance from the scattering centre, we find that at a distance 1.4 nm (i.e., the observed distance between nearest neighbouring FeFPc and MnPc species on the Au(111) substrate), the molecular spins are expected to couple antiferromagnetically. Furthermore, next-nearest neighbours at a distance of 1.98 nm are expected to couple ferromagnetically. If we, however, assume the same distances between magnetic centres of a FeFPc+MnPc checkerboard array, but assembled on a Ag(111) substrate, the RKKY interaction would lead to a ferromagnetic

coupling between nearest neighbours and weak antiferromagnetic coupling for next-nearest neighbours. Thus, the above given argument and the *ab initio* calculations presented in the main text support that the long-range ferrimagnetic order is mediated by the RKKY coupling via the Au substrate.

Recently, for tetracyano-p-quinodimethane (TCNQ) molecules assembled on graphene/Ru(0001) the existence of a ferromagnetic ordered ground state with an in-plane moment orientation was claimed on the basis of spin polarised STM/STS measurements performed on different monomolecular domains[15]. However, the magnetic contrast which was resolved only after averaging across small areas of differently oriented domains on rather noisy data, exhibits a contrast between parts of the sample that does not discriminate long-range ferromagnetism from paramagnetism with disordered patches of TCNQ molecules having magnetisation either up or down, and is hence of limited evidence for establishing long-range order in the system. Furthermore, the proposed direct Heisenberg exchange mechanism[15] via the intermolecular hybridisation between frontier orbitals of the TCNQ molecules would not be able to lead to long-range ferromagnetic order in a truly 2D system at finite temperatures, as this would contradict the Mermin-Wagner theorem.

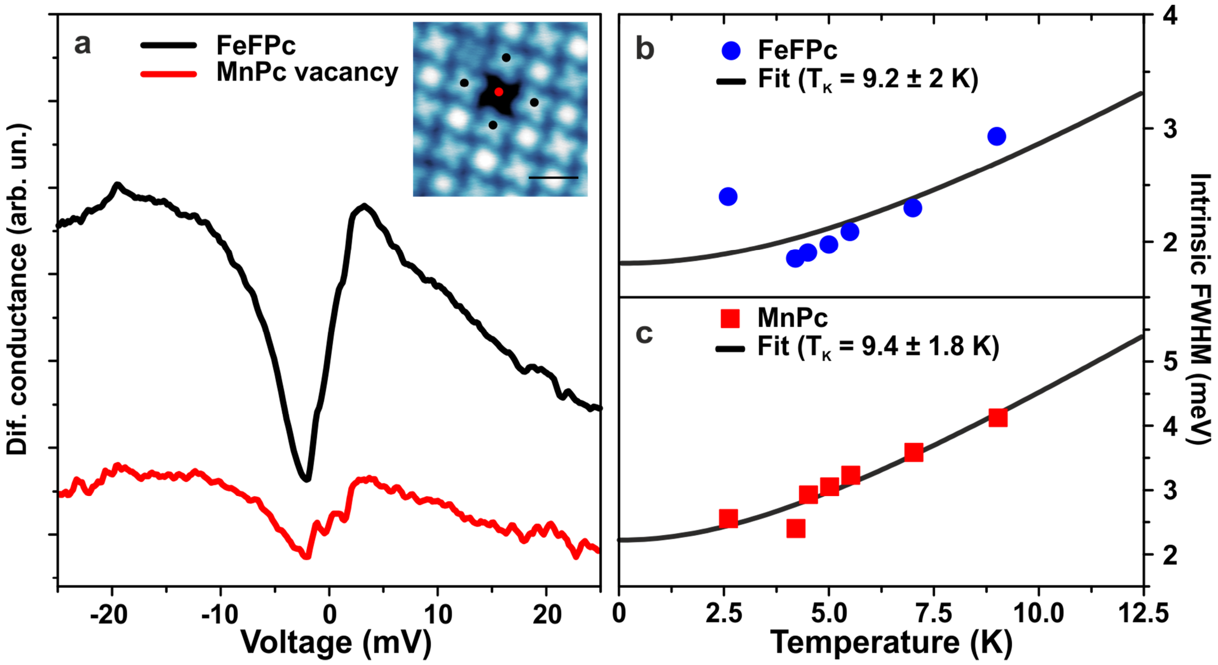

Supplementary Figure 5 | Results of STS measurements. (**a**) STS measurements taken on an MnPc vacancy within the supramolecular layer (inset). The scale bar in the inset is 2 nm. The STS spectra were recorded in the centre of the vacancy (red dot) and on centres of neighbouring FeFPc molecules (black dots). (**b,c**) Kondo temperatures of MnPc and FeFPc on Au(111) extracted from a fit of the Kondo resonance in the Fermi-liquid model. The statistical error in the Kondo temperature is a root-mean-square deviation given by the fit to the Frota function [Supplementary Eq. (5)].

Supplementary Note 4.

In this Supplementary Note we report the measurement of the Kondo temperatures of MnPc and FeFPc molecules in the supramolecular array as well as scanning tunnelling spectroscopy on a MnPc vacancy.

Scanning tunnelling spectroscopy recorded on a missing MnPc molecule of the supramolecular monolayer displays the so-called "cloning" of the zero-bias features of the neighbouring FeFPc molecules (see **Supplementary Figure 5a**). Such a "cloning" of the Kondo state present on neighbouring atoms adsorbed on a metallic substrate has been previously demonstrated for Co atoms adsorbed on Cu(111) by placing the atom in one of the focal points in the artificially constructed quantum corrals[16,17]. In our case, the Kondo signature measured in the centre of a MnPc vacancy is replicated without the need of an additionally constructed confined state. The observed effect readily demonstrates that the Au(111) surface states are weakly affected by the adsorbed molecules and consequently that these surface states play an important role in mediating the RKKY exchange coupling between MnPc and FeFPc molecules. This observation is in good agreement with a previous report, where a "cloning" was observed for a missing FePc molecule in a 2-dimensional FePc lattice adsorbed on Au(111)[18]. The here-measured Kondo temperature of the MnPc molecules is slightly higher T_K = 9.4±1.8 K than the one of the FeFPc species T_K = 9.2±2 K (see the fits in **Supplementary Fig. 5b,c**).

The Kondo temperatures have been obtained by measuring scanning tunnelling spectra at the centre of the magnetic molecules at different temperatures. The STS spectra have subsequently been fitted with the Frota function[19] in the form given by Prüser *et al.* [20]:

$$\frac{\mathrm{d}I}{\mathrm{d}V} \sim a * \mathrm{Im}\left[ie^{i\varphi}\sqrt{\frac{i\Gamma}{eV - e_0 + i\Gamma}}\right] + b * V + c \quad (5)$$

where φ is phase factor defining the shape of the Kondo zero bias feature, Γ stands for a resonance width, e_0 is centre of the Kondo resonance and a, b, c are multiplication factors and a constant, respectively. The temperature dependent width of the Kondo resonance, extracted from the fit to function in supplementary equation (5) has been fitted with the Fermi-liquid model [Supplementary Eq. (6)]. The Kondo temperature was extracted from the fit that models the resonance width as a function of the temperature (see **Supplementary Fig. 5b,c**) employing the expression[21,22]

$$\Gamma = \sqrt{(\alpha k_\mathrm{B} T_\mathrm{eff})^2 + (2k_\mathrm{B} T_\mathrm{K})^2} \quad (6)$$

$$T_\mathrm{eff} = \frac{1}{5.4k_\mathrm{B}}\sqrt{(5.4k_\mathrm{B}T)^2 + (1.7V_\mathrm{rms})^2} \quad (7)$$

where V_rms is the root mean square of modulation voltage, T_K the Kondo temperature, k_B is the Boltzmann constant and α is a multiplication factor defining the slope of the curve at $T \gg T_\mathrm{K}$.

In the fitting process we obtained the following parameters: α = 4.55 ± 0.33 and φ = 1.69±0.1 for MnPc and α = 2.58± 0.65 and φ = 4.29±0.045 for FeFPc.

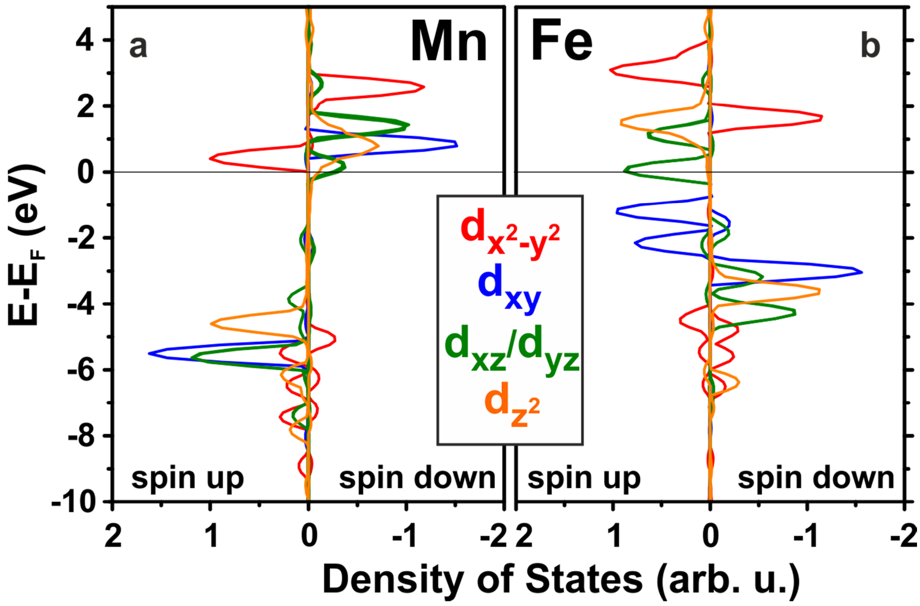

Supplementary Figure 6 | *Ab initio* calculated partial density of states (DOS). (**a**) The partial DOS of the 3d orbitals of Mn and (**b**) of Fe within FeFPc and MnPc molecules assembled on Au(111), computed with the DFT+*U* method.

Supplementary Note 5.

The electronic structure of the FeFPc+MnPc supramolecular monolayer on Au(111) has been computed within the density-functional theory +*U* (DFT+*U*) framework. In the open 3d-shell of the central metal ion of the metallo-phthalocyanine there are strong on-site d-d Coulomb correlations, which are captured by the supplemented Hubbard *U* and on-site exchange constant *J* (supplementary ref. 23). The importance of the Coulomb correlations has been shown previously for a number of metallo-porphyrins and metallo-phthalocyanines; in particular, they lead to molecular magnetic moments and spin-crossover temperatures that are significantly improved[24-26] over those given by standard density functional theory (DFT) calculations. In the present calculations *U* and *J* were taken to be 4 eV and 1 eV, respectively. These values were shown to provide the correct spin state for free as well as substrate absorbed metallo-phthalocyanines and metallo-porphyrins[27,28]. For the DFT exchange-correlation functional we used the generalised-gradient approximation (GGA) in the parameterisation of Perdew *et al.*[29]. The electronic structure calculations were performed with the VASP full-potential plane-wave code[30] with a kinetic energy cut-off of 400 eV.

As we find that the molecules are relatively weakly adsorbed on the Au(111) surface with an adsorption energy of 0.88 eV per molecule (see Methods) there could be an influence of the Van der Waals interaction. To investigate this we performed calculations with the Van der Waals-Density Functional (vdW-DF) method[31]. We find however that the vdW-DF calculations predict a significantly larger lattice constant for Au (4.245 Å, as compared to the experimental value of 4.08 Å). As this larger Au atomic distance would have an influence on the adsorbed molecules within the self-consistent relaxation process of the atomic distances, we decided to use only the DFT+*U* framework with the GGA exchange-correlation description.

We further mention that our STM images show the so-called herringbone reconstruction of the Au surface (see **Fig. 1c**). This reconstruction occurs on a length scale of about 20 nm, which is much larger than the sizes of our simulation cell (laterally 28.85 x 29.98 Å^2). Hence, the herringbone reconstruction has not been taken into account in our simulations. However, the important length scale for the indirect exchange interaction is the distance between the FeFPc and MnPc molecules, which is much smaller, about 14 Å. Therefore the herringbone reconstruction is expected not to influence this exchange interaction noticeably.

The computed 3d spin moments were -2.1 μ_B and 3.7 μ_B, for FeFPc and MnPc, respectively, on Au(111). The computed 3d moments with the Au substrate removed were very similar, 2.0 μ_B and 3.6 μ_B, respectively. These values correspond to S = 1 for FeFPc and $S \approx 3/2 - 2$ for MnPc. The spin-resolved local 3d-densities of states (DOS) of the central Fe and Mn ions, calculated with the DFT+U approach are shown in **Supplementary Fig. 6**. These partial densities give the following orbital populations of the 3d metal ions: $(d_{xy})^2\,(d_{xz},d_{yz})^{2.6}(d_{z^2})^1(d_{x^2-y^2})^{0.7}$ (i.e., $(b_{2g})^2\,(e_g)^{2.6}(a_{1g})^1(b_{1g})^{0.7}$) for FeFPc, and $(d_{xy})^1\,(d_{xz},d_{yz})^2(d_{z^2})^1(d_{x^2-y^2})^{0.8}$ for MnPc, where the population of the $d_{x^2-y^2}$ orbital is responsible for a reduction of the total spin S.

Upon adsorption of FeFPc and MnPc on Au(111) we find that the molecular geometry slightly changes. In the gas phase, the FeFPc and MnPc molecules have D_{4h} symmetry, but adsorbed on the Au(111) surface MnPc adopts C_{4v} and FeFPc C_{2v}. In case of FeFPc two of its benzene rings moved slightly towards the substrate, while in case of MnPc the phthalocyanine plane came closer to the substrate yet with the Mn atom positioned 0.17 Å above the macrocyclic ring.

To investigate whether the selfconsistently computed antiparallel alignment of moments on the FeFPc and MnPc molecules represents the lowest energy state, we have carried out additional total energy calculations for two other magnetic phases. Specifically, we calculated the ferromagnetic (FM) phase (all Fe and Mn moments are parallel) and a different ferrimagnetic (AFM1) phase, where one of the Fe moments was reversed, enforcing thus an antiferromagnetic alignment on the FeFPc sublattice. The selfconsistently calculated total energies are given in **Supplementary Table 2**. We note that in these different magnetic states the same molecular structure has been used, in order to extract purely the magnetic interaction.

Supplementary Table 2. Results of DFT+U calculations performed for different magnetic arrangements of the FeFPc+MnPc supramolecular layer on Au(111). AFM0 refers to the ferri-magnetic state where the spin moments on FeFPc and MnPc are antiparallel. FM refers to the ferromagnetic state with parallel-aligned spin moments on the Fe and Mn atoms. AFM1 refers to a different ferrimagnetic state, in which the spin moment on one of the Fe-atoms has been reversed, to be parallel to the Mn-moments.

Magnetic phase	FM	AFM1	AFM0
Total energy E_{PBE+U} [eV]	-2789.15291242	-2789.15359549	-2789.15447126
Magnetic moments μ_{Fe1}, μ_{Fe2}, μ_{Mn1}, μ_{Mn2} [μ_B]	2.02, 2.02, 3.69, 3.69	-2.02, 2.02, 3.69, 3.69	-2.02, -2.02, 3.69, 3.69

The total energy calculations confirm that the AFM0 state with antiparallel orientations of the Fe and Mn moments is the ground state, in agreement with our XMCD measurements. In addition we observe that the ferromagnetic state has a total energy that is higher by 1.5588 meV per 2x2 unit cell, or 0.78 meV per structural unit cell (containing one FeFPc and one MnPc molecule), which corresponds to 9 K. The AFM1 state with one flipped Fe magnetic moment has a total energy that is higher than that of AFM0 by 0.876 meV per 2x2 unit cell, or 5 K per structural unit cell. These calculated exchange interactions are expectedly very small, which is consistent with the weak mediation of the long-range exchange RKKY interaction via the Au substrate. The calculated exchange energies are in good agreement with the long-range magnetic ordering temperature of about 5 K estimated from our temperature-dependent XMCD measurements. Assuming a nearest-neighbour-only exchange interaction Hamiltonian, $H = 8J_{\mathrm{Fe-Mn}}S_{\mathrm{Fe}}S_{\mathrm{Mn}}$, provides a calculated value $J_{\mathrm{Fe-Mn}} = 0.10$ meV, which agrees well with the strength of the exchange interaction estimated from the XMCD versus magnetic field measurements.

Supplementary References

1. Stöhr, J. & König, H. Determination of spin- and orbital-moment anisotropies in transition metals by angle-dependent X-ray magnetic circular dichroism. *Phys. Rev. Lett.* **75**, 3748–3751 (1995).
2. Bernien, M. X-Ray Absorption Spectroscopy of Fe Complexes on Surfaces: Electronic Interactions and Tailoring of the Magnetic Coupling. *PhD Thesis, Free University Berlin* (2009).
3. Bartolomé, J. *et al.* Highly unquenched orbital moment in textured Fe-phthalocyanine thin films. *Phys. Rev. B* **81**, 195405 (2010).
4. Thole, B. T., Carra, P., Sette, F. & van der Laan, G. X-ray circular dichroism as a probe of orbital magnetization. *Phys. Rev. Lett.* **68**, 1943–1946 (1992).
5. Carra, P., Thole, B. T., Altarelli, M. & Wang, X. X-ray circular dichroism and local magnetic fields. *Phys. Rev. Lett.* **70**, 694–697 (1993).
6. Piamonteze, C., Miedema, P. & de Groot, F. M. F. Accuracy of the spin sum rule in XMCD for the transition-metal L edges from manganese to copper. *Phys. Rev. B* **80**, 184410 (2009).
7. Teramura, Y., Tanaka, A. & Jo, T. Effect of Coulomb interaction on the X-ray magnetic circular dichroism spin sum rule in 3d transition elements. *J. Phys. Soc. Jpn.* **65**, 1053–1055 (1996).
8. Crocombette, J. P., Thole, B. T. & Jollet, F. The importance of the magnetic dipole term in magneto-circular x-ray absorption dichroism for 3d transition metal compounds. *J. Phys. Condens. Mater.* **8**, 4095–4105 (1996).
9. Hermanns, C. F., *et al.* Huge magnetically coupled orbital moments of Co porphyrin molecules and their control by CO adsorption. *Phys. Rev. B* **88**, 104420 (2013).
10. Stepanow, S. *et al.* Mixed-valence behavior and strong correlation effects of metal phthalocyanines adsorbed on metals. *Phys. Rev. B* **83**, 220401 (2011).
11. Lisi, S. *et al.* Graphene-induced magnetic anisotropy of a two-dimensional iron phthalocyanine network. *J. Phys. Chem. Lett.* **6**, 1690–1695 (2015).
12. Bruno, P. Tight-binding approach to the orbital magnetic moment and magnetocrystalline anisotropy of transition-metal monolayers. *Phys. Rev. B* **39**, 865(R)–868(R) (1989).
13. Reinert, F., Nicolay, G., Schmidt, S., Ehm, D., & Hüfner, S. Direct measurements of the L-gap surface states on the (111) face of noble metals by photoelectron spectroscopy. *Phys. Rev. B* **63**, 115415 (2001).
14. Fischer, B. & Klein, M. W. Magnetic and nonmagnetic impurities in two-dimensional metals. *Phys. Rev. B* **11**, 2025–2029 (1975).
15. Garnica, M. *et al.* Long-range magnetic order in a purely organic 2D layer adsorbed on epitaxial graphene. *Nat. Phys.* **9**, 368–374 (2013).
16. Manoharan, H. C., Lutz, C. P. & Eigler, D. M. Quantum mirages formed by coherent projection of electronic structure. *Nature* **403**, 512–515 (2000).
17. Crommie, M. F., Lutz, C. P. & Eigler, D. M. Confinement of electrons to quantum corrals on a metal surface. *Science* **262**, 218–220 (1993).
18. Tsukahara, N. *et al.* Evolution of Kondo resonance from a single impurity molecule to the two-dimensional lattice. *Phys. Rev. Lett.* **106**, 187201 (2011).
19. Frota, H. O. Shape of the Kondo resonance. *Phys. Rev. B* **45**, 1096–1099 (1992).
20. Prüser, H. *et al.* Interplay between the Kondo effect and the Ruderman–Kittel–Kasuya–Yosida interaction. *Nat. Commun.* **5**, 5417 (2014).
21. Otte, A. F. *et al.* The role of magnetic anisotropy in the Kondo effect. *Nat. Phys.* **4**, 847–850 (2008).
22. Nagaoka, K., Jamneala, T., Grobis, M. & Crommie, M. F. Temperature Dependence of a Single Kondo Impurity. *Phys. Rev. Lett.* **88**, 077205 (2002).
23. Anisimov, V. I., Aryasetiawan, F. & Lichtenstein, A. I. First-principles calculations of the electronic structure and spectra of strongly correlated systems: the LDA + U method. *J. Phys. Condens. Matter* **9**, 767–808 (1997).

24. Panchmatia, P. M., Sanyal, B. & Oppeneer, P. M. GGA + U modeling of structural, electronic, and magnetic properties of iron porphyrin-type molecules. *Chem. Phys.* **343**, 47–60 (2008).
25. Ali, M. E., Sanyal, B. & Oppeneer, P. M. Electronic structure, spin-states, and spin-crossover reaction of heme-related Fe-porphyrins: A theoretical perspective. *J. Phys. Chem. B* **116**, 5849–5859 (2012).
26. Maldonado, P., Kanungo, S., Saha-Dasgupta, T. & Oppeneer, P. M. Two-step spin-switchable tetranuclear Fe(II) molecular solid: Ab initio theory and predictions. *Phys. Rev. B* **88**, 020408 (2013).
27. Wäckerlin, C. *et al.* On-surface coordination chemistry of planar molecular spin systems: novel magnetochemical effects induced by axial ligands. *Chem. Sci.* **3**, 3154–3160 (2012).
28. Hermanns, C. F. *et al.* Magnetic coupling of porphyrin molecules through graphene. *Adv. Mater.* **25**, 3473–3477 (2013).
29. Perdew, J. P., Burke, K. & Ernzerhof, M. Generalized gradient approximation made simple. *Phys. Rev. Lett.* **77**, 3865–3868 (1996).
30. Kresse, G. & Furthmüller, J. Efficiency of ab-initio total energy calculations for metals and semiconductors using a plane-wave basis set. *Comput. Mater. Sci.* **6**, 15–50 (1996).
31. Berland, K., *et al.* Van der Waals forces in density functional theory: a review of the vdW-DF method. *Rep. Prog. Phys*. **78**, 066501 (2015).
